# Engineering Microbial Particles for Next‐Generation Biomedical Platforms

**DOI:** 10.1002/advs.202600012

**Published:** 2026-04-17

**Authors:** Yuting Li, Yunjie Lu, Ziyan Huang, Jianjiang Chen, Yanbin Liu, Lei Qin, Chao Wang, Yumin Wu

**Affiliations:** ^1^ Department of Hepatobiliary Surgery Department of General Surgery The First Affiliated Hospital of Soochow University Suzhou China; ^2^ Institute of Functional Nano & Soft Materials (FUNSOM) Soochow University Suzhou Jiangsu China; ^3^ State Key Laboratory of Targeting Oncology Guangxi Medical University Nanning Guangxi China; ^4^ Department of Neurology The First Affiliated Hospital of Soochow University Suzhou Jiangsu China; ^5^ Biomedical Basic Research Center (BBRC) of Jiangsu Province Suzhou Jiangsu China

**Keywords:** biomedical engineering, intelligent biological platform, microbe‐derived particles

## Abstract

Microbe‐derived particles (MDPs), which include extracellular vesicles, outer membrane vesicles, inclusion bodies, polysaccharide particles, and virus‐like particles, represent a rapidly expanding category of bioinspired nanomaterials. With their natural origin, intrinsic biocompatibility, and highly programmable functionality, MDPs serve as a versatile bridge between living microbial systems and engineered nanomaterials. This review systematically outlines the diverse classes, biosynthetic pathways, physicochemical properties, and biomedical applications of MDPs. We summarize recent progress in their use for drug delivery, vaccine design, immune modulation, and diagnostic imaging, emphasizing how bioengineering approaches enable precise control over their composition and targeting capabilities. Additionally, we examine advancing methodologies for scalable manufacturing and accurate characterization—such as omics‐based profiling and advanced imaging techniques. Finally, we discuss current challenges and future prospects for integrating synthetic biology with materials science to develop next‐generation intelligent biological platforms based on MDPs. This comprehensive overview aims to foster the rational design and translational deployment of MDP‐based systems across biomedical, biotechnological, and materials science fields.

## Graphical abstract

Microbe‐derived particles (MDPs), which include extracellular vesicles, outer membrane vesicles, inclusion bodies, polysaccharide particles, and virus‐like particles, represent a rapidly expanding category of bioinspired nanomaterials. With their natural origin, intrinsic biocompatibility, and highly programmable functionality, MDPs serve as a versatile bridge between living microbial systems and engineered nanomaterials. This review systematically outlines the diverse classes, biosynthetic pathways, physicochemical properties, and biomedical applications of MDPs. We summarize recent progress in their use for drug delivery, vaccine design, immune modulation, and diagnostic imaging, emphasizing how bioengineering approaches enable precise control over their composition and targeting capabilities. Additionally, we examine advancing methodologies for scalable manufacturing and accurate characterization¡^a^such as omics‐based profiling and advanced imaging techniques. Finally, we discuss current challenges and future prospects for integrating synthetic biology with materials science to develop next‐generation intelligent biological platforms based on MDPs. This comprehensive overview aims to foster the rational design and translational deployment of MDP‐based systems across biomedical, biotechnological, and materials science fields.

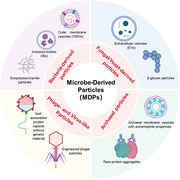

## 1 Introduction

Over the past two decades, the field of biomedical nanotechnology has undergone a paradigm transition—from the design of *artificial nanomaterials* to the creation of *bioinspired and bio‐derived systems*. The early generation of synthetic nanoparticles, including metallic, polymeric, and inorganic systems, provided programmable physicochemical properties that revolutionized drug delivery, imaging, and immunomodulation [1, 2, 3]. However, despite their precision in design, these materials often face biological bottlenecks such as rapid immune clearance, limited biodegradability, and insufficient physiological adaptability, which hinder their translational potential [4, 5].

Cell‐derived biomaterials, typically defined as bioactive materials fabricated from living cells or their derivatives such as extracellular vesicles, possess inherent advantages due to their cellular origin, including intrinsic targeting capability, low immunogenicity, and physiologically relevant molecular heterogeneity [6, 7]. Among these, microbe‐derived particles (MDPs) represent a major class of biologically grounded nanoplatforms. MDPs differ fundamentally from mammalian EVs in their origin and composition: unlike EVs, which are secreted by eukaryotic cells and primarily carry host‐derived signaling proteins, lipids, and RNAs, MDPs originate from microorganisms and inherently contain microbial‐specific components such as cell wall fragments, pathogen‐associated molecular patterns (PAMPs), and microbial enzymes. These features confer unique immunogenicity, biophysical properties, and interaction modes with host systems. In contrast to fully synthetic nanoparticles, MDPs combine intrinsic biological functionality with the potential for genetic and modular engineering, allowing programmable cargo loading, surface modification, and tunable bioactivity. Additionally, compared to mammalian cell‐derived particles, MDPs offer practical advantages, including easier scalability for mass production, more straightforward genetic manipulation, and lower cost, making them highly promising platforms for immune modulation and other biomedical applications. Originating from a phylogenetically diverse range of microorganisms, including Gram‐negative/Gram‐positive bacteria, filamentous fungi, and extremophilic archaea, microbial extracellular particles manifest as a heterogeneous family of nanostructures with distinct biophysical and biochemical signatures (Table 1). This architectural diversity is exemplified by: (1) outer membrane vesicles (OMVs), bilayered nanostructures (20–200 nm) shed from the outer membrane of Gram‐negative bacteria, which inherently package lipopolysaccharides (LPS), outer membrane proteins (OMPs), and periplasmic enzymes; (2) inclusion bodies (IBs), amorphous or crystalline proteinaceous aggregates (50–500 nm) formed in bacterial/fungal cytoplasm during recombinant protein overexpression, characterized by high stability and intrinsic affinity for biomacromolecular cargos, (3) virus‐like particles (VLPs), non‐infectious nanostructures (20–100 nm) self‐assembled from viral structural proteins n engineered microbes, mimicking viral morphology without containing replicative genetic material, and (4) extracellular polysaccharide particles (EPPs), nanocomposites (100–1000 nm) composed of microbial exopolysaccharides and associated proteins, exhibiting high biocompatibility and mucoadhesive properties [8, 9, 10]. Their advantages include biomanufacturing scalability, tunable immunogenicity, and genetic programmability, making them powerful tools for therapeutic vaccination, cancer immunotherapy, microbial sensing, and bioremediation [11, 12, 13].

**TABLE 1:** Comparison of composition, characteristics, and applications of particles from various microbial sources.

Type	Source	Main Composition	Size Range (nm/µm)	Surface / Physicochemical Features	Stability / Characteristics	Modifiable Sites	Typical Applications
OMVs	Gram‐negative bacteria	Phospholipids, LPS, outer membrane proteins	20–200 nm	Naturally secreted; high immunogenicity; drug‐loading capability	Moderate	Amine, carboxyl	Vaccines, immunotherapy, and delivery systems
IBs	Prokaryotes	Recombinant protein aggregates	50–500 nm	High purity; controllable release; sustained‐release behavior	High	Carboxyl, thiol	Protein storage; sustained‐release carriers
EPS	Bacteria / Fungi	Polysaccharide complexes	50 nm–several µm	Strong adhesion; high biocompatibility	High	Carboxyl, hydroxyl	Biofilm formation; drug encapsulation
Fungal EVs	Yeast / Fungi	Lipids, proteins, RNA	50–300 nm	Immune interaction with host; pressure‐resistant stability	Moderate	Phospholipid head groups	Infection markers; vaccine candidates
β‐glucan particles	Yeast	β‐1,3/1,6‐glucan	100–1000 nm (1–5 µm)	Immune activation; drug‐carrying potential	High	Hydroxyl	Immune modulation; drug delivery
VLPs	Viruses / Phages	Capsid proteins	20–100 nm	Non‐genetic; self‐assembly precision	High	Exposed amino acid residues	Vaccines; mRNA delivery; imaging
Archaeal EVs (aEVs)	Archaea	Phospholipids, proteins	50–400 nm	Thermostable; low immunogenicity	High	—	Extreme‐environment carriers; industrial applications

Synthetic biology and molecular engineering have further enabled modular control over the composition, cargo loading, and surface display of MDPs, transforming them from passive byproducts into engineered, multifunctional bio‐nanoplatforms. By incorporating engineered gene circuits, rewired vesiculation or secretion pathways, and rationally designed membrane‐anchoring domains, microorganisms can be directed to fine‐tune the molecular constituents of the particles they release, allowing selective enrichment of nucleic acids, proteins, metabolites, or therapeutic agents within MDPs, as well as precise surface customization through the display of targeting ligands, immune modulators, or receptor‐mimetic motifs. Together, these multilayered engineering strategies effectively convert MDPs from incidental microbial byproducts into purpose‐built, multifunctional bio‐nanoplatforms with highly tunable properties, underscoring their potential to address unmet needs in precision medicine, environmental protection, and industrial biotechnology.

**FIGURE 1:**
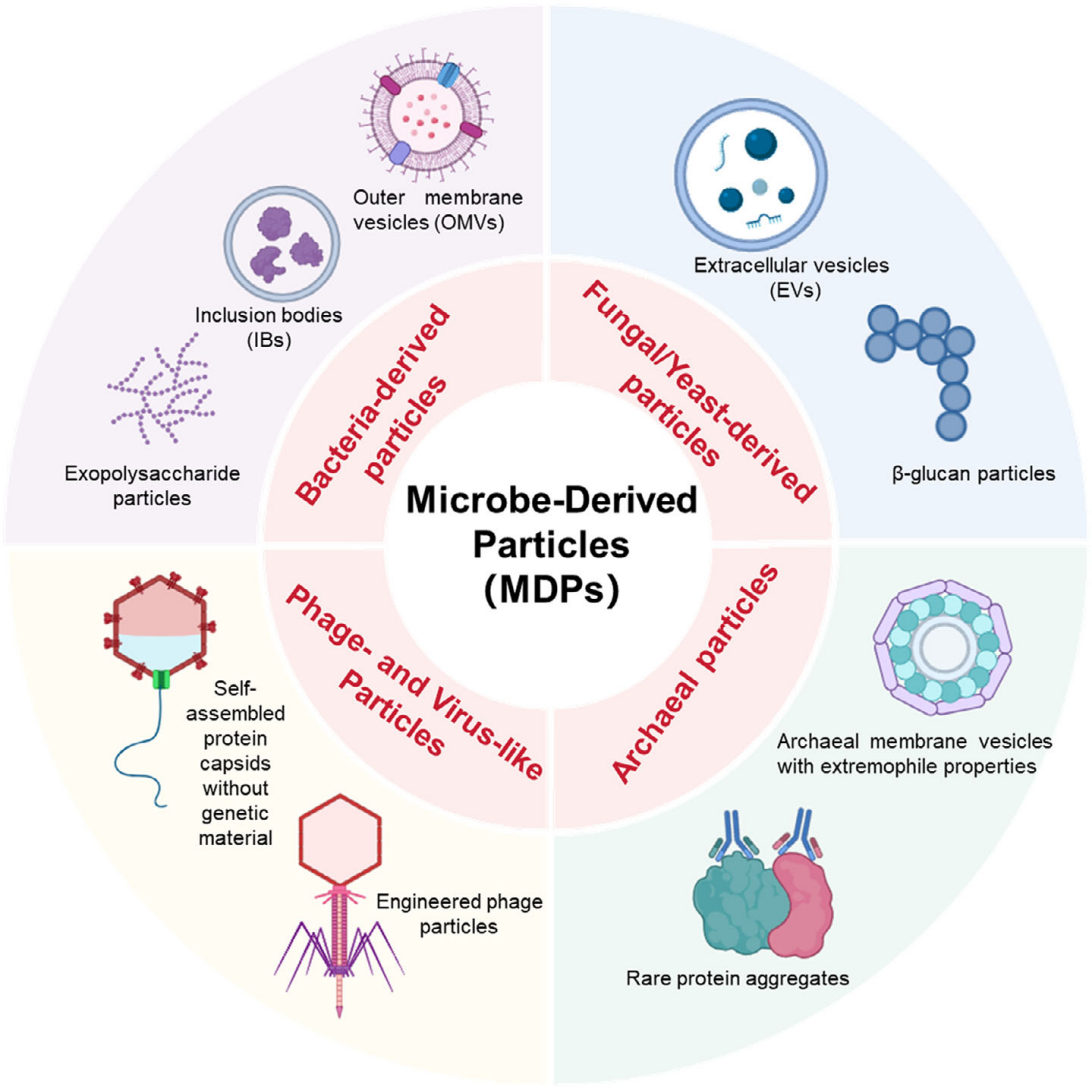
Diversity and hierarchical organization of MDPs. MDPs exhibit extensive structural and functional diversity across different biological origins. Bacteria‐derived particles include OMVs, IBs, and EPS granules, typically serving as immunogenic carriers or structural scaffolds. Fungal‐ and yeast‐derived particles mainly comprise EVs and β‐glucan particles, which mediate intercellular communication and immune modulation. Phage and VLPs are genome‐free self‐assembled protein shells that can be engineered for vaccine and delivery applications. Archaea‐derived vesicles and protein aggregates demonstrate remarkable stability and adaptation to extreme environments. Together, these natural and engineered microbial particles constitute a hierarchical bio‐nanomaterial system with broad potential for biomedical, environmental, and biotechnological applications.

In this review, we provide a comprehensive overview of the origins, biological architectures, biosynthetic mechanisms, and engineering strategies of MDPs, emphasizing their unique position within the landscape of bioinspired nanotechnology. We further discuss their biomedical and biotechnological applications, including immune modulation, targeted delivery, and environmental defense, as well as the key challenges that must be overcome to achieve clinical translation and regulatory standardization. Ultimately, we propose that MDPs represent not merely a new class of nanomaterials but a biological paradigm of programmable particulate systems, where microbial evolution, molecular engineering, and synthetic biology converge to inspire the next generation of intelligent living therapeutics (Figure 1) [14, 15, 16].

## 2 Classification of Microbial‐Source Particles

### 2.1 Bacterial Source Particles

#### 2.1.1 Outer Membrane Vesicles

Outer membrane vesicles are nanoscale vesicular structures naturally released by Gram‐negative bacteria during growth through a controlled outer membrane blebbing process [17, 18]. Their discovery dates back to the 1960s. Early studies regarded OMVs as by products of membrane instability; however, accumulating evidence has since demonstrated that OMV production is a ubiquitous and highly regulated physiological phenomenon, representing a major secretory pathway of bacteria [19].

The mechanisms of OMV biogenesis can be broadly classified into two categories. The first involves outer membrane blebbing triggered by local imbalances in peptidoglycan cross‐linking, lipid redistribution, or insertion of hydrophobic molecules into the bilayer. The second arises from phage endolysins or autolysins that induce explosive cell lysis, giving rise to outer–inner membrane vesicles (OIMVs) with dual membrane structures and explosive outer membrane vesicles (EOMVs) [20, 21] are spherical bilayer structures [22, 23], they display morphological uniformity and structural stability, appearing as intact spherical vesicles under transmission electron microscopy. Some bacterial species can also form continuous chains of OMVs or connect OMVs to nanotubes, thereby facilitating intercellular exchange of materials [24]. OMV production has been well‐documented across diverse Gram‐negative bacteria species, including *Escherichia coli*, *Pseudomonas aeruginosa*, *Vibrio cholerae*, *Helicobacter pylori*, and *Salmonella Typhimurium* [25, 26]. Recent studies have revealed that some Gram‐positive bacteria species, *actinomycetes*, and *mycoplasmas* also generate analogous membranous vesicles (e.g., cytoplasmic membrane vesicles, CMVs), suggesting that vesicle biogenesis is a conserved physiological feature among microorganisms [27, 28].

The composition of OMVs is complex and heterogeneous, encompassing lipids, proteins, nucleic acids, and small metabolites. Notably, the outer layer is enriched with LPS, lipoproteins, and outer membrane proteins, whereas the lumen can encapsulate virulence factors, enzymes, signaling molecules, and fragments of DNA, RNA, or plasmids [29]. The cargo varies with their biogenesis pathway: classical OMVs generated via membrane blebbing are enriched in outer membrane‐associated components, whereas OIMVs and EOMVs derived from cell lysis may also contain cytoplasmic proteins and nucleic acids. The selective packaging of these molecules reflects bacterial strategies to adapt to environmental stresses and physiological states [30].

Functionally and translationally, OMVs play essential roles in bacterial ecological adaptation and pathogenesis, while also exhibiting promising biomedical applications. Their inherent immunogenicity and delivery capacity render them attractive vaccine platforms, as exemplified by the clinically approved meningococcal OMV vaccine. Engineered OMVs can also serve as delivery vehicles for specific therapeutics or anticancer agents [31, 32]. Moreover, their membrane architecture and biocompatibility position them as ideal building blocks for bio‐nanomaterials and diagnostic platforms. Taken together, OMVs represent a universally conserved system of intercellular communication and cargo transfer shaped by bacterial evolution. Their mechanisms of formation, compositional diversity, and functional versatility hold profound implications for ecology, infection biology, and bioengineering [33, 34].

#### 2.1.2 Inclusion Bodies

Inclusion bodies are insoluble protein aggregates formed in cells under conditions of high‐level heterologous protein expression or cellular stress [35]. First identified in *Escherichia coli* via electron microscopy in the 1980s, IBs were initially regarded as protein misfolding by‐products (“waste materials”) that impaired recombinant protein expression efficiency [36]. However, advances in understanding protein folding mechanisms and structural biology have revealed that IBs not only contain misfolded proteins but also frequently retain partial native‐like conformations, secondary structures, and even catalytic activity [37]. Consequently, IBs have gradually shifted from being seen as problematic by‐products to valuable resources [38].

Morphologically, IBs are typically localized at cell poles or in the perimembrane region, appearing as spherical or rod‐shaped particles with diameters ranging from 0.5 to 1 µm [39, 40]. They display a porous architecture and certain degrees of hydration. Their cores are enriched with high concentrations of the protein of interest, accompanied by small amounts of lipids, nucleic acids, and molecular chaperones such as DnaK and GroEL. These chaperones facilitate protein aggregation and local folding. Additionally, IBs possess high mechanical strength, thermal stability, and resistance to proteolytic degradation, which provide advantages in downstream purification and handling from both operational and economic perspectives.

In terms of origin, IBs are predominantly observed in prokaryotic systems, particularly in *Escherichia coli expression* hosts. This is primarily attributed to the reducing environment of the cytoplasm, the absence of sophisticated chaperone networks, and the rapid translation rates, all of which favor folding imbalances and aggregation. IBs can also form in yeast, insect, and mammalian cells when heterologous proteins are overexpressed, although their yields and aggregation characteristics often differ from those in prokaryotic hosts [41]. Compared to the densely packed IBs in prokaryotic hosts, which are usually highly abundant and largely misfolded, IBs in eukaryotic cells form at lower levels, exhibit a looser structure, and often retain a fraction of correctly folded or functional proteins.

Compositionally, IBs are primarily composed of misfolded or partially folded proteins, frequently enriched in β‐sheet structures without full denaturation. Recent studies have demonstrated that many proteins within IBs retain reversible refolding potential, and some even function as directly usable catalytic particles. The applications of IBs have become increasingly prominent. First, they act as efficient intermediates for recombinant protein production and purification. In industrial fermentations, enable protein recovery via washing, solubilization with chaotropic agents (e.g., urea, SDS, or guanidine hydrochloride), and refolding to obtain high‐purity soluble products. Second, they serve as functional bionanomaterials with potential applications for tissue engineering scaffolds, drug delivery carriers, and enzyme immobilization. Third, inherent active IBs are directly used in industrial biocatalysis; Their high antigen density and particulate nature make them valuable for vaccine/immunotherapy research, such as IBs of HPV16 major capsid proteins in virus‐like particle (VLP) vaccine studies. [42, 43].

Despite these advantages, challenges remain in the preparation and refolding of IBs. Conventional processes often depend heavily on empirical optimization, leading to low refolding yields and poor reproducibility. Humer and Spadiut (2018) emphasized the importance of integrating Process Analytical Technology (PAT) and Quality by Design (QbD) principles into IB processing [44]. Specifically, techniques such as dynamic light scattering (DLS), circular dichroism (CD), attenuated total reflectance Fourier‐transform infrared spectroscopy (ATR‐FTIR), Raman spectroscopy, and nuclear magnetic resonance (NMR) can provide real‐time or offline monitoring to improve process control. Moreover, fed‐batch refolding strategies have been shown to enhance efficiency and space‐time yield, rendering IB processing more controllable and industrially viable.

In summary, IBs are naturally occurring nanoscale protein aggregates formed under high expression stress. They possess complex structures and versatile functions. IBs not only represent the limits of cellular protein‐folding homeostasis but also provide an efficient form of protein storage and transformation for biomanufacturing. With deeper insights into their formation mechanisms, refolding kinetics, and process monitoring strategies, IBs are evolving from passive by‐products to designable, controllable, and utilizable functional biomaterials, with broad application prospects in recombinant protein production, industrial catalysis, drug delivery, and vaccine development.

#### 2.1.3 Exopolysaccharide Particles

Bacterial exopolysaccharides (EPSs) are high‐molecular‐weight carbohydrate polymers synthesized and secreted by microorganisms into the extracellular environment. Since Sutherland first systematically described them in 1972 [45], EPS research has expanded considerably. Compared to polysaccharides derived from plants, animals, or algae, bacterial EPSs offer significant advantages in terms of controllable production, rapid synthesis, and independence from seasonal or climatic limitations. Based on their association with the bacterial cell surface, EPSs are classified into capsular polysaccharides, which are tightly bound to the cell surface, and slime polysaccharides, which are loosely secreted into the surrounding environment. Both types serve as the primary matrix of biofilms, affording protection to microbial communities. EPSs exhibit high hydrophilic, biodegradable, and amenable to chemical modification [46, 47].

A wide range of bacteria produce EPSs, including Gram‐negative genera such as *Xanthomonas* and *Pseudomonas*, as well as Gram‐positive genera such as *Bacillus* and *Lactobacillus*. EPSs can be further divided into homopolysaccharides (e.g., dextran, bacterial cellulose) and heteropolysaccharides (e.g., xanthan, alginate), depending on their monosaccharide composition [48, 49]. Biologically, EPSs are essential for bacterial survival and ecological adaptation: they enable cells to withstand extreme temperature, salinity, desiccation, pH fluctuations, and antibiotic pressure. Moreover, EPSs facilitate cell adhesion, aggregation, and biofilm formation, thereby enhancing resistance to antimicrobial agents. In pathogenic bacteria, EPSs play important roles in virulence regulation, while in symbiotic systems, they contribute to community stability by acting as a “molecular glue” [50, 51].

**FIGURE 2:**
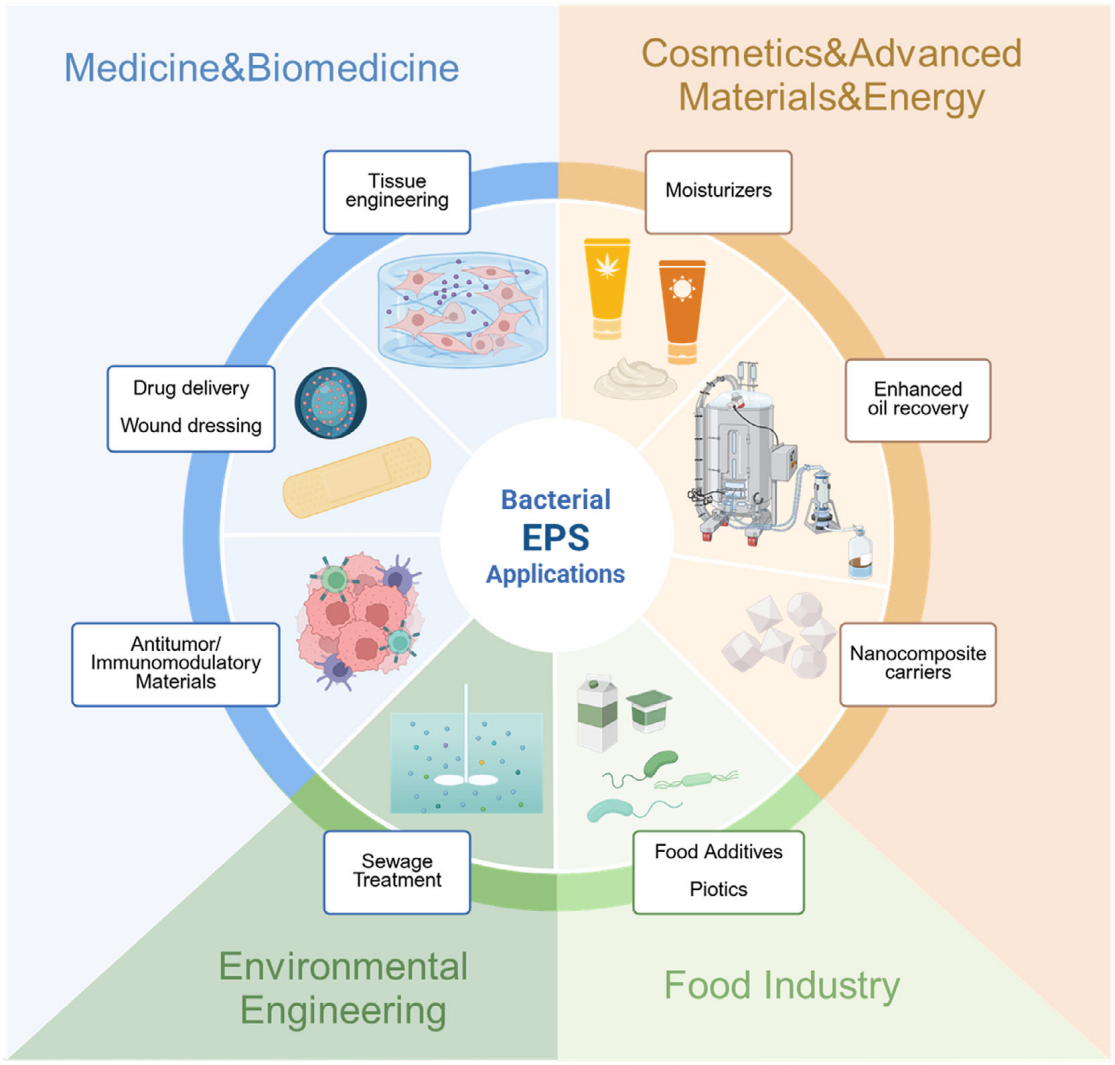
Schematic illustration of the diverse applications of bacterial extracellular polysaccharides (EPS). The central core highlights the fundamental properties of EPS—biocompatibility, biodegradability, non‐toxicity, and tunable structural properties—that enable their widespread use. The surrounding sectors detail specific applications across four key industries: Medicine & Biomedicine (blue), including scaffolds for tissue engineering, carriers for drug delivery, wound dressings, and antitumor/immunomodulatory materials; Environmental Engineering (dark green), illustrating wastewater treatment via biofiltration of heavy metals and organic pollutants; the Food Industry (light green), demonstrating uses as thickeners, emulsifiers, stabilizers, and prebiotics; and Cosmetics, Advanced Materials, & Energy (yellow/orange), showing applications such as moisturizers, nanocomposite carriers, and enhanced oil recovery.

Because of their unique biocompatibility, biodegradability, non‐toxicity, and tunable structural properties, bacterial EPSs exhibit enormous application potential (Figure 2). In the food industry, they are widely employed as thickeners, emulsifiers, stabilizers, and prebiotics [52, 53]. In medicine and biomedicine, EPSs have been further developed as scaffolds for tissue engineering, carriers for drug and growth factor delivery, wound dressings, and functional materials with antitumor and immunomodulatory activities [54]. In environmental engineering, EPSs are effective biosorbents for heavy metals and organic pollutants, and thus are applied in wastewater treatment [55]. Furthermore, EPSs are utilized in cosmetics, advanced material science, and energy industries, for example, as moisturizers, carriers for functional nanocomposites, and biocatalytic matrices to enhance oil recovery [56, 57, 58].

Despite these remarkable properties, the range of EPSs that have reached industrial‐scale production remains very limited. The major bottlenecks lie in the relatively low yields of producing strains and the high production costs [59, 60]. Future progress will rely on advances in metabolic engineering and synthetic biology, including strain optimization, the development of low‐cost media (e.g., based on agro‐industrial waste), and the exploration of biomimetic catalytic strategies such as quorum‐sensing (QS)‐based approaches, to unlock the full potential of bacterial EPSs in green manufacturing, biomedical materials, and environmental remediation. Additionally, exploration of novel EPSs from extremophiles (e.g., marine microorganisms), combined with non‐culture‐based techniques such as metagenomics, will be key to expanding the application boundaries of EPSs [61, 62].

### 2.2 Fungal/Bacterial Source Particles

#### 2.2.1 Fungal Extracellular Vesicles

Fungal extracellular vesicles (EVs) are nanoscale, membrane‐enclosed structures actively secreted by fungi. The earliest observation of such vesicular particles can be traced back to 1958, when “extramembranous particles” were identified in the wood‐decaying fungus *Coriolus versicolor* [63, 64, 65]. However, their existence was not firmly confirmed until 2007, when studies on *Cryptococcus neoformans* demonstrated that polysaccharides were exported via vesicular compartments, thereby confirming both the presence of fungal EVs and their function in mediating trans‐cell wall transport [64, 66].

The biogenesis of fungal EVs involves at least two distinct pathways. In the endosomal pathway, multivesicular bodies (MVBs) fuse with the plasma membrane and release exosome‐like vesicles into the extracellular space. In parallel, the plasma membrane budding pathway generates microvesicle‐like structures through direct outward protrusions of the plasma membrane [67, 68]. These vesicles typically range from 20 to 200 nm in diameter, exhibit a buoyant density of 1.10–1.25 g/mL, and display the characteristic bilayer membrane structure. Notably, some fungal EVs are coated with fibrillar polysaccharide extensions—a feature that may facilitate their biological interactions.

Fungal EVs have been reported across a wide range of species throughout the fungal kingdom. They are produced not only by human pathogenic fungi, such as C. neoformans and Candida albicans, but also by important plant pathogens, including Botrytis cinerea and Magnaporthe oryzae, as well as model organisms like *Saccharomyces cerevisiae* and *Aspergillus nidulans*. The molecular composition of these vesicles is remarkably diverse, incorporating proteins such as Hsp70 and chitinases, polysaccharides including glucuronoxylomannan (GXM) and β‐glucan, nucleic acids such as small RNAs and messenger RNAs, and distinctive fungal lipids such as ergosterol [69, 70].

Beyond their structural and compositional characteristics, fungal EVs have emerged as functional mediators in both pathogenesis and host interactions. In medical research, EVs derived from *C. neoformans* have been shown to elicit protective immune responses, leading to a significant improvement in host survival, with murine experiments reporting up to a 60% increase in survival rates following vaccination. In agricultural contexts, plant‐derived EVs have been exploited as delivery systems for small interfering RNAs that target genes associated with fungal EV function [71], thereby effectively reducing the incidence of diseases such as gray mold caused by *B. cinerea*. From a broader biological perspective, fungal EVs have revealed novel modes of cross‐kingdom communication, particularly through the delivery of fungal small RNAs that suppress host immune gene expression, thereby providing a mechanistic explanation for certain host–microbe interactions.

Despite these advances, major challenges continue to impede the progress of fungal EV research. A universal classification system has yet to be established, and the precise mechanisms by which these vesicles traverse the rigid fungal cell wall remain unresolved. Furthermore, the lack of specific biomarkers for fungal EVs has hindered in vivo functional studies. Recent proposals, such as the use of tetraspanin PLS1 as a candidate EV marker, highlight ongoing efforts to develop reliable molecular tools, which are expected to facilitate a deeper understanding of fungal EV biology in the near future.

#### 2.2.2 β‐Glucan Particles

β‐Glucans are polysaccharides widely distributed in fungi, yeasts, algae, lichens, and some bacteria. As structural components of fungal cell walls, they play a key role in maintaining cell wall integrity [72, 73]. The biosynthesis of β‐glucans is mediated by β‐1,3‐glucan synthases, such as the FKS1‐Rho1 complex in yeast. Since these enzymes are absent in human cells, they have also emerged as important targets for antifungal therapeutics [74]. Recent studies have successfully isolated β‐glucans from diverse sources, including the pathogenic fungus *Candida albicans*, the common yeast *Saccharomyces cerevisiae* [75, 76], and edible mushrooms such as *Grifola Frondosa* [77], *Agaricus bisporus*, and *Trametes versicolor*, and have prepared structurally intact β‐glucan particles, commonly referred to as whole‐glucan particles (WGPs) [78, 79].

Morphologically, β‐glucan particles characteristically exhibit a hollow “ghost cell” structure, predominantly constituted by the β‐glucan layer of the cell wall—a structural feature that endows them with inherent porosity and robust structural stability. Notably, the internal domain of these particles often adopts a triple‐helical secondary structure, a conserved conformational trait that confers substantial structural stability and distinct physicochemical properties critical for their biological functions [80, 81, 82]. Furthermore, the molecular weight and solubility vary depending on the source: yeast‐derived particles are generally high‐molecular‐weight and insoluble, mushroom‐derived β‐glucans often possess highly polymerized β‐1,3/β‐1,6 linkages, and algal β‐glucans such as laminarin are low‐molecular‐weight and soluble [83, 84].

In terms of applications, β‐glucan particles have attracted attention due to their distinctive structure and immunomodulatory activity. They can act as ligands for the immune receptor Dectin‐1, activating innate immune cells and inducing trained immunity, thereby enhancing host resistance to subsequent infections [85]. In addition, β‐glucan particles demonstrate notable antioxidant and antitumor activities; when loaded with bioactive compounds such as quercetin, they can achieve sustained release and enhanced anticancer effects, showing promising inhibitory activity in models such as prostate cancer [86, 87]. Furthermore, β‐glucans have been reported to regulate lipid metabolism and improve cholesterol levels, indicating potential cardiovascular protective effects and broad applicability in functional foods and nutraceuticals [88, 89]. The hollow, porous structure of β‐glucan particles also confers advantages for drug delivery, enabling controlled release and targeted delivery [90, 91]. Finally, since β‐1,3‐glucan synthase is absent in humans, related research has also promoted the development of antifungal drugs targeting it [92].

In summary, since their discovery, β‐glucan particles have gradually emerged as an important research focus at the intersection of mycology, immunology, and pharmaceutical applications. Their unique molecular architecture and broad natural sources provide a solid foundation for applications in immunomodulation, antitumor therapy, drug delivery, and the development of functional foods.

### 2.3 Bacteriophages and Virus‐Like Particles

#### 2.3.1 Genome‐Free Self‐Assembling Protein Capsids

Genome‐free self‐assembling protein capsids, also termed VLPs, are nanoscale particles derived from bacteriophages [93]. Their discovery originates from foundational structural and assembly studies of RNA bacteriophage MS2 and DNA bacteriophage T5 [94]. Specifically, MS2 VLPs are formed through the self‐assembly of 180 coat protein (CP) subunits into a T = 3 icosahedral structure with a diameter of approximately 22–29 nm. The stability of these particles is modulated by environmental factors, including temperature, pH, and ionic strength, and can be enhanced through targeted mutations. In contrast, the empty capsid‐like particles (CLPs) of bacteriophage T5 are composed of 775 pb8 protein subunits arranged in an expanded 90 nm structure, with the surface further decorated by 120 pb10 proteins or their fusion variants through extremely high‐affinity interactions (KD ≈ 10^−^^1^ ^2^ m) [95, 96].

The assembly of these particles is independent of genetic material. Specifically, MS2 VLPs can form through specific interactions between coat proteins and RNA pac sites or through spontaneous self‐assembly in the absence of RNA templates, whereas T5 CLPs achieve efficient surface decoration through in vitro maturation and autonomous anchoring of the pb10 domain (pN). Morphologically, both systems exhibit highly ordered repetitive antigen arrays, remarkable thermal stability (T5 CLPs withstand up to 95°C), and resistance to proteases and nucleases.

In terms of applications, MS2 and T5 VLPs have been developed as versatile delivery platforms. MS2 VLPs possess the capacity to encapsulate diverse cargo molecules, including mRNA, miRNA, peptides, or small molecules for use in vaccine development (e.g., prostate cancer mRNA vaccines, FMDV peptide vaccines), gene therapy (e.g., miR‐146a delivery to modulate immune responses), and diagnostic standards (armored RNA/DNA) [97, 98]. In contrast, T5 CLPs achieve targeted antigen presentation through genetic fusion of target antigens to pb10 proteins, which efficiently enables the surface display of large antigens such as ovalbumen. This strategy elicits potent and durable adaptive immune responses, encompassing humoral (multiple IgG subclasses) and CD8^+^ cytotoxic T cell responses even in the absence of exogenous adjuvants. This observation highlights the intrinsic adjuvant properties of T5 CLPs and their promising potential for targeted antigen delivery in immunotherapeutic applications [99].

Both systems exemplify the advantages of modular design. Specifically, MS2 VLPs support genetic fusion or chemical conjugation, while T5 CLPs exploit the high‐affinity pN domain to achieve directional antigen presentation. Their biocompatibility, scalability, and low immunotoxicity further promote their applications in biomedical fields, including infectious disease control, cancer immunotherapy, and standardized molecular diagnostics. Future research should focus on optimizing antigen compatibility, particularly for eukaryotic post‐translational modifications, and improving in vivo targeting efficiency to fully realize the clinical potential of these biomimetic nanomaterials.

#### 2.3.2 Engineered Bacteriophage Particles

The discovery and development of engineered bacteriophage particles and VLPs represent significant milestones in microbiology and virology, reflecting the progression from fundamental findings to engineered applications. Bacteriophages were first independently discovered by Frederick Twort in 1915 and Félix d'Hérelle in 1917 [100, 101, 102], a landmark revelation that demonstrated the specificity of viral parasitism on bacteria and laid the groundwork for subsequent bacteriophage research. In the 1980s, Smith successfully inserted foreign DNA fragments encoding peptides into the pIII coat protein gene of the filamentous phage f1, enabling the surface display of exogenous peptides and establishing the foundational phage display technology [103]. This approach not only became a critical tool for molecular screening but also initiated the use of bacteriophages as engineered delivery vectors. The research on VLPs originated from detailed viral structural analyses. Early VLPs were employed for atomic‐level structural elucidation of viruses [104, 105]. With the advent of heterologous expression technologies, researchers demonstrated that the core protein (HBc) and surface antigen (HBsAg) of the hepatitis B virus (HBV) could self‐assemble into VLPs in heterologous systems [106, 107, 108, 109]. The first HBV VLP‐based vaccine was developed by Merck in 1981 using HBsAg‐VLPs, and the recombinant human hepatitis B vaccine received approval in 1986, marking VLPs as an established platform for vaccine development. More recently, engineering modifications of bacteriophages have expanded their functional applications; for instance, λ phages engineered via homologous recombination and Cas13a counter‐selection have been used to deliver CRISPR transposase systems, enabling precise editing of microbial communities—highlighting the expanding utility of phage‐based tools in synthetic biology and microbiome engineering [110, 111].

Morphologically, engineered bacteriophage particles and VLPs exhibit highly structured architectures, which can be precisely tuned through engineering to meet specific application requirements. Bacteriophages display remarkable morphological diversity, primarily categorized as filamentous or icosahedral. The filamentous phage M13, approximately 880 nm in length and 6–7 nm in diameter, can have its particle length adjusted by controlling the length of encapsulated nucleic acids. In icosahedral phages [112, 113, 114], T7 possesses a 55 nm icosahedral capsid containing 40 kbp linear double‐stranded DNA with a short, non‐contractile tail of ∼19 nm, whereas T4 is larger, with an elongated icosahedral head (120 nm long and 86 nm wide) enclosing 171 kbp linear double‐stranded DNA and a contractile tail equipped with six ∼160 nm long tail fibers critical for host recognition [115]. VLPs closely mimic natural viruses in morphology, typically forming icosahedral or helical structures with diameters ranging from 20 to 200 nm, enabling efficient lymphatic drainage and interaction with antigen‐presenting cells (APCs) and B cells [116]. For example, HPV L1 protein self‐assembles into VLPs with a 55 nm icosahedral structure, closely resembling native HPV virions, while MS2 coat protein forms 26 nm icosahedral VLPs with precise symmetry. Advanced engineering approaches, such as introducing disulfide bonds at coat protein pentamer interfaces, have further improved assembly fidelity, generating uniform T = 1 icosahedral VLPs at nanometer‐scale precision.

The biological origins of engineered bacteriophage particles and VLPs differ fundamentally, a distinction that dictates their production paradigms and application scopes. Bacteriophages, as viruses with host specificity, are intimately linked to their bacterial hosts, with different types infecting specific bacterial strains. For instance, M13, T7, and T4 phages naturally infect Escherichia coli; M13 is a temperate phage that releases progeny without lysing the host, while λ phage exhibits both lytic and lysogenic lifecycles, and its engineered variants, such as those carrying the Sam7 amber mutation, restrict lysis to amber‐suppressor hosts, refining host specificity. In contrast, VLPs are derived from viral structural proteins and do not require complete viruses, enabling their production in various heterologous systems, including bacteria (E.coli), yeast (Saccharomyces cerevisiae), mammalian cells (HEK293), insect cells, and plants. Typical examples include HBV, HPV, and HEV structural proteins, which self‐assemble into VLPs, and MS2 VLPs, derived from bacteriophage coat proteins expressed in heterologous systems. Different expression systems offer trade‐offs; bacterial systems are low‐cost and high‐yield but lack post‐translational modifications, whereas mammalian systems support complex modifications at a higher cost [117, 118].

In terms of composition, both engineered bacteriophage particles and VLPs are protein‐based but differ in nucleic acid content and functional cargo loading capabilities, which can be customized through engineering. Bacteriophages consist of nucleic acids and coat proteins; their genomes may be linear or circular double‐stranded DNA (e.g., T7, T4, λ phages) or single‐stranded RNA (e.g., MS2), carrying essential replication information. Coat proteins are divided into major structural subunits, forming the phage body (e.g., ∼2700 copies of M13 pVIII, T7 gp10A/gp10B), and minor proteins involved in host recognition or assembly (e.g., M13 pIII, T4 HOC and SOC). Functional elements can be incorporated by replacing non‐essential genomic regions, such as λ‐DART phages, where a 12.3 kb non‐essential region is replaced with a DART transposon system encoding CRISPR‐associated transposases and transposon sequences. VLPs, by contrast, lack genomes and replication machinery, relying solely on viral structural proteins such as HBc, HBsAg, HPV L1, or MS2 coat proteins. Functional cargo can be engineered into VLPs, for instance, MS2 VLPs selectively package exogenous RNA via a 19‐nucleotide pac site or display antigenic peptides in the AB loop. Directed evolution strategies, exemplified by the fifth‐generation v5 eVLPs, introduce mutations (e.g., Q226P, C507V) in coat proteins to optimize ribonucleoprotein cargo packaging and release efficiency [119, 120].

### 2.4 Archaeal‐Derived Particles

#### 2.4.1 Vesicles with Adaptation to Extreme Environments

Extracellular Vesicles (EVs) with Adaptation to Extreme Environments are primarily produced by archaea, microorganisms widely distributed in extreme habitats such as high‐temperature, high‐pressure, high‐salinity, or acidic environments [121, 122]. The discovery of archaeal EVs has deepened our understanding of the adaptive mechanisms of life. The biogenesis of these vesicles is closely linked to the unique archaeal cell membrane structure, which is composed of ether‐linked isoprenoid chains, such as glycerol‐1‐phosphate backbone lipids [123]. This configuration significantly enhances membrane stability under high‐temperature and high‐pressure conditions. Some archaea, such as *Ignicoccus hospitalis*, possess a complex double‐membrane architecture, which even allows spatial separation of energy metabolism processes, potentially representing an evolutionary prototype of early cellular membrane systems [124].

Morphologically, archaeal EVs are typically spherical, with diameters ranging from 100 to 300 nm and an average size of approximately 130 nm, comparable to bacterial EVs. Certain species, such as *Pyrodictium*, can form tubular connections (cannulae), while *Altiarchaeum hamiconexum* produces hook‐like appendages (hami) that facilitate cell attachment and biofilm formation [125, 126]. These diverse morphologies reflect functional specialization and environmental adaptation.

The producing organisms include hyperthermophilic archaea (e.g., *Thermococcus*, *Sulfolobus*), halophilic archaea (e.g., *Haloferax*), and methanogenic archaea in the human gut (e.g., *Methanobrevibacter smithii*, *Methanosphaera stadtmanae*), which release vesicles to cope with environmental pressures in their respective niches. Compositionally, archaeal EVs are enriched in characteristic lipids, such as diether or tetraether lipids. Proteomic analyses reveal a high abundance of adhesin‐like proteins (ALPs) with immunoglobulin‐like (Ig‐like) domains, likely mediating cell recognition and attachment [127, 128]. Additionally, these vesicles carry various metabolic enzymes and transport proteins, such as oligopeptide transporters (OPT), as well as free amino acids (e.g., glutamate, aspartate) and signaling molecules (e.g., glycerophosphocholine), which may participate in intercellular communication, proton gradient maintenance, and stress response [129].

#### 2.4.2 Rare Protein Aggregates

Initially observed in extremophilic archaea, such as *Thermococcales* and *Sulfolobales*, the formation of these aggregates is likely linked to the unique archaeal cell envelope structure, including membranes composed of surface layer (S‐layer) proteins and ether lipids [130, 131]. Recent studies have demonstrated that commensal archaea in the human gut, including *Methanobrevibacter smithii*, *M. intestini*, and *Methanosphaera stadtmanae*, also continuously produce archaeal extracellular vesicles (AEVs) during growth [132].

Morphologically, AEVs appear as membrane‐enclosed spherical structures with an average diameter of approximately 130 nm and a size range of 50–400 nm, similar to bacterial extracellular vesicles (BEVs). However, their concentration is typically lower, ranging from 1.4 × 10^10^ to 3.7 × 10^1^ ^1^ particles/mL [133, 134]. Electron microscopy reveals that these vesicles can be attached to the archaeal cell surface, located intracellularly, or freely suspended in the culture medium, displaying well‐defined boundaries. Proteomic analysis shows that AEVs are enriched in a class of adhesins or adhesin‐like proteins (ALPs), comprising up to 20% of total vesicle protein. These proteins often contain immunoglobulin‐like (Ig‐like) domains or pectinase‐like folds, suggesting key roles in mediating interactions between archaea, bacteria, or host cells [135]. Additionally, AEVs contain oligopeptide transporters (OPT superfamily), DUF11‐domain proteins, and a variety of hydrolases. Metabolomic studies further reveal selective enrichment of free amino acids such as glutamate and aspartate, as well as neuroactive molecules including glycerophosphocholine, implying potential involvement in host physiological regulation via the gut–brain axis [136].

Functional investigations indicate that AEVs can be efficiently internalized by human macrophages and induce intestinal epithelial cells (e.g., HT‐29) and immune cells (e.g., THP‐1‐derived macrophages) to produce specific cytokines and chemokines, including CXCL9 [137, 138], CXCL11 [139, 140], IL‐8 [141, 142], and CX3CL1 [143], demonstrating their immunomodulatory potential. Given their nanoscale size, biocompatibility, and cargo of bioactive molecules, AEVs hold considerable promise as novel drug delivery vehicles, modulators of inflammatory diseases, or microbiome engineering tools. Future research should aim to elucidate their biogenesis mechanisms, functional roles in complex intestinal environments, and feasibility for clinical translation.

Each class of MDP offers unique structural and functional attributes. OMVs provide strong inherent immunogenicity and versatile surface display but may require endotoxin mitigation. Inclusion bodies are highly stable with excellent cargo‐loading capacity, yet generally lack innate targeting. Virus‐like particles feature precise architecture and programmability, though their production is technically demanding. Extracellular polysaccharide particles are biocompatible and mucoadhesive but can suffer from heterogeneity and purification challenges. Recognizing these relative strengths and limitations is essential for selecting the most suitable particle type for specific biomedical or biotechnological applications.

## 3 Characteristics of Microbe‐Derived Particles

### 3.1 Intrinsic Bioactivity

**FIGURE 3:**
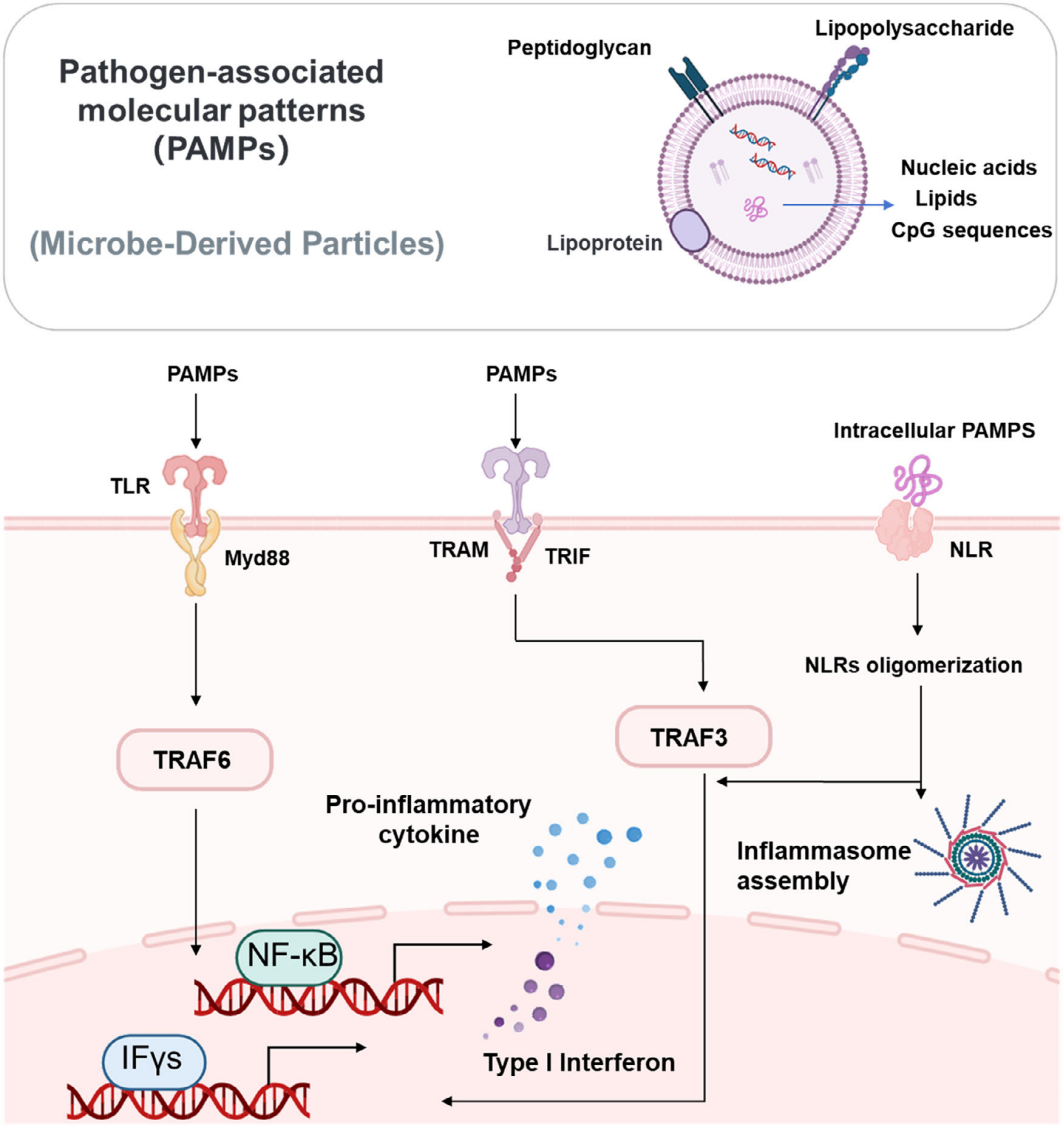
Schematic of host innate immune recognition triggered by PAMPs carried by MDPs. MDPs are intrinsically enriched with diverse PAMPs on their surface and within their interior, which can be precisely detected by host PRRs. Surface‐exposed lipopolysaccharides, lipoproteins, and peptidoglycans activate membrane‐associated receptors such as TLR4 and TLR2, leading to NF‐κB and IRF3 activation via MyD88‐ or TRIF‐dependent signaling, thereby inducing the expression of pro‐inflammatory cytokines and antiviral genes. Intracellular components released upon particle uptake can be sensed by NOD‐like receptors, promoting their oligomerization and inflammasome assembly, which drives maturation of inflammatory mediators. Together, these complementary recognition pathways endow MDPs with potent innate immune‐activating capacity.

A central advantage of microbial‐derived particles is their innate carriage of biologically active motifs, notably PAMPs (Figure 3) [144, 145], which fundamentally shape immunogenicity, chemotactic behavior, and receptor‐mediated targeting. Representative PAMPs include lipopolysaccharide (LPS), lipoproteins, peptidoglycan (PG), lipid A variants, β‐glucans, and nucleic acids enriched in unmethylated CpG motifs [146]. These motifs engage pattern recognition receptors (PRRs) — e.g., TLR4 for LPS, TLR2 for lipoproteins/PG, Dectin‐1 for β‐glucans, and NOD1/NOD2 for PG fragments — triggering NF‐κB/IRF signaling that culminates in proinflammatory cytokines (TNF‐α, IL‐6, IL‐1β), chemokines, and costimulatory molecule expression, thus bridging innate and adaptive immunity [89, 147, 148, 149, 150].

For particle platforms, PAMPs exert a double‐edged influence. On one hand, they confer self‐adjuvanting properties, enhancing dendritic cell (DC) activation, antigen cross‐presentation, and cytotoxic CD8^+^ T‐cell responses — a desirable attribute for vaccines and immunotherapies. Engineered OMVs delivering tumor‐associated antigens exemplify this, simultaneously providing antigen payloads and innate immune stimulation, thereby reducing the need for exogenous adjuvants [19, 23]. On the other hand, uncontrolled or excessive PAMP activity (e.g., unmodified lipid A) can provoke systemic inflammation or endotoxic shock, constraining safe in vivo dosages and clinical translation. Therefore, modulating PAMP identity, abundance, and activity — e.g., via lipid A modification or msbB knockout — is central to engineering safer microbial particles [151].

Distinct particle classes exhibit characteristic immune signatures: bacterial OMVs commonly drive strong innate inflammation and Th1 polarization; fungal β‐glucan–containing particles stimulate Dectin‐1–mediated Th17 and phagocytic responses supportive of antifungal and antitumor immunity [152]; VLPs with repetitive epitope arrays potently induce humoral (B cell) responses and neutralizing antibodies [153]. Cargo nucleic acids within particles may also trigger RIG‐I/MDA5 pathways, generating antiviral interferon programs that enhance vaccine efficacy [154].

Thus, harnessing intrinsic bioactivity while mitigating adverse inflammatory outcomes is the design principle for effective microbial‐derived nanoparticle platforms.

### 3.2 Assembly Mechanisms: Self‐Assembly, Multilayered Membrane Structures, and Protein Folding‐Driven Formation

**FIGURE 4:**
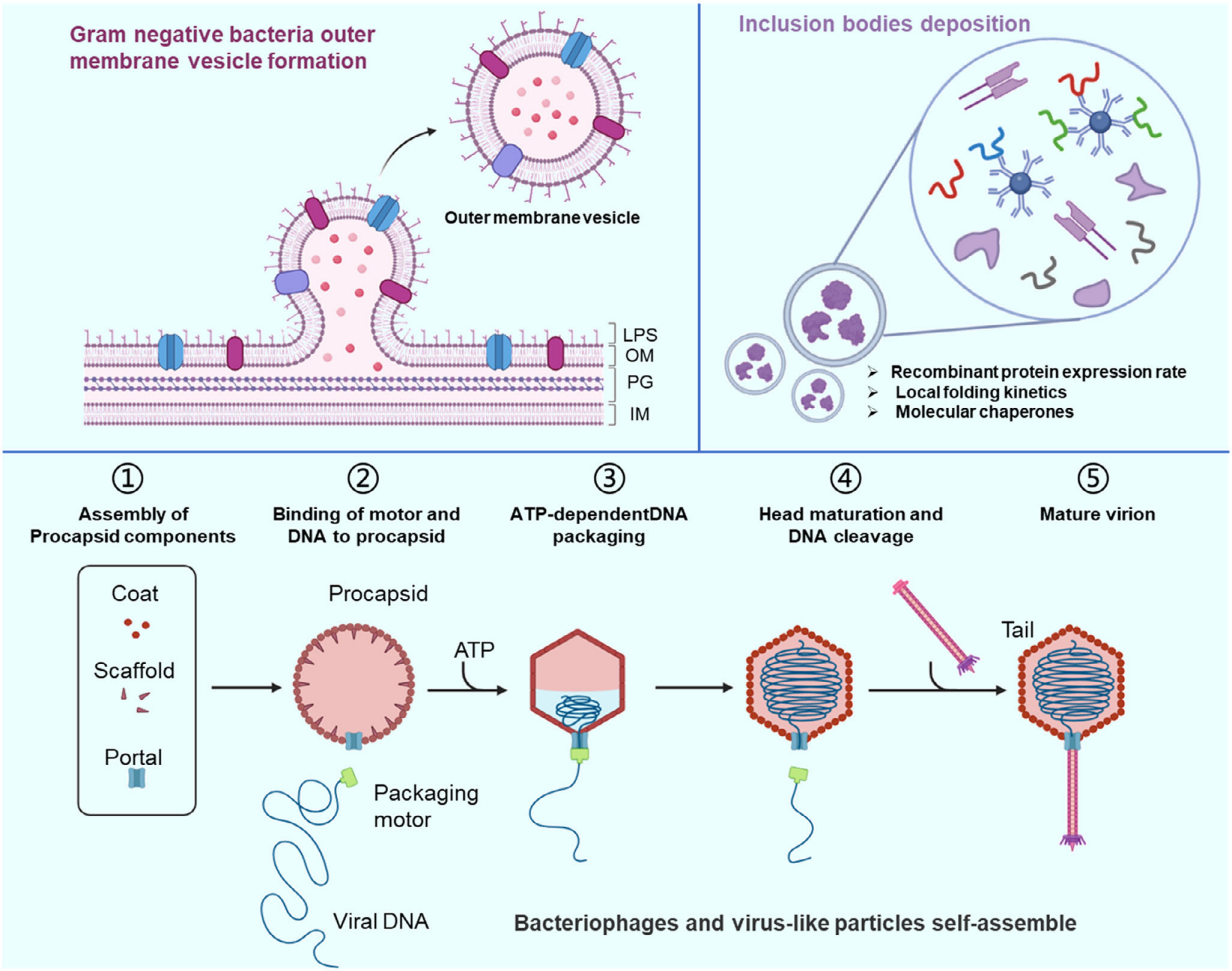
Distinct assembly mechanisms of biological particles. OMVs are produced by budding from the outer membrane of Gram‐negative bacteria, representing a self‐assembly process driven by membrane curvature and lipid–protein interactions. IBs arise from the deposition of recombinant proteins due to imbalanced expression rate, local folding kinetics, and limited assistance from molecular chaperones. In contrast, VLPs and bacteriophages undergo a highly ordered self‐assembly cascade involving procapsid formation, binding of motor and DNA to the procapsid, ATP‐dependent genome packaging, and head maturation, ultimately yielding infectious or structurally complete virions.

The biogenesis of microbial particles stems from several fundamental physicochemical drivers: membrane curvature and asymmetry–induced budding, protein‐protein interaction–driven self‐assembly, and aggregation driven by misfolding or multivalent interactions (Figure 4). Mastery of these drivers is essential to control particle size, homogeneity, and functional presentation.

OMV budding is tightly linked to membrane lipid composition, accumulation of particular membrane proteins, and the state of anchoring to the peptidoglycan layer. Disruption of protein–peptidoglycan linkages (e.g., through differential expression of OmpA, Lpp) or asymmetric lipid distribution can produce local curvature stress and vesiculation [29]. Environmental stressors (antibiotics, pH shifts) often upregulate vesiculation, indicating adaptive control points amenable to engineering.

VLP and phage capsid assembly exemplify protein self‐organization: capsid subunits interact via specific interfaces, hydrophobic cores, and electrostatic complements to form symmetric shells (icosahedral, T‐numbers), with assembly sensitive to ionic strength, pH, and cofactors [155]. Molecular chaperones or assembly factors often modulate assembly kinetics and fidelity.

Inclusion body (IB) formation results from expression overload and folding kinetics; exposure of hydrophobic segments leads to non‐specific aggregation, often forming ordered nanofibers or crystalline lattices [156, 157, 158]. By tuning induction temperature, co‐expressing chaperones, or incorporating controllable aggregation tags (e.g., elastin‐like peptides), IB size, hydrophobic exposure, and release profiles can be regulated [159].

Moreover, multilayered or multicompartment vesicles can be assembled using differential lipid affinities and membrane remodeling mechanisms — valuable for partitioned cargo delivery (hydrophilic vs hydrophobic compartments). Microfluidic and in vitro reconstitution platforms allow precise fabrication of such multicompartment architectures, enabling sophisticated functional integration [160].

For engineers, the central takeaway is that manipulating expression intensity, lipid composition, environmental parameters, and auxiliary factors (chaperones/assembly peptides) provides handles to tune particle yield, size distribution, and cargo localization, thereby enabling programmable production and downstream functionalization.

### 3.3 Physicochemical Properties: Nano‐ to Micrometer‐Sized Dimensions, Stability, Surface Charge, and Modifiability

The physicochemical properties of microbial particles critically determine their in vitro and in vivo behaviors. Key properties include particle size & morphology, stability, surface charge & hydration, and chemical modifiability. Size and morphology: Sizes range from ∼20 nm (small VLPs, small OMVs) to micron scales (large IBs, β‐glucan particles). Size influences lymphatic drainage, uptake pathways, and immunogenicity: 10–100 nm particles preferentially traffic to lymph nodes and promote B‐cell responses, whereas 100–1000 nm particles are more readily phagocytosed, promoting antigen presentation and T‐cell immunity. Application goals (humoral vs cellular immunity) thus guide size selection (Table 2).

**TABLE 2:** Key PAMPs, Receptors, and Immune Outcomes.

Particle class	Dominant PAMPs	Recognized PRRs	Dominant immune outcome	Engineering handles
OMVs	LPS, lipoproteins	TLR4, TLR2	DC maturation, Th1 skewing	Lipid A modification, OMP fusions
VLPs	Repetitive capsid epitopes	TLR7/9 (if RNA), BCR crosslinking	Strong B cell / neutralizing Ab	Epitope insertion, chemical conjugation
β‐glucan particles	β‐glucan	Dectin‐1	Phagocytosis, Th17/innate activation	Size tuning, surface loading
IBs	Protein aggregates	Phagocytic uptake	APC activation (if presented)	Fusion tags, partial refolding

Stability (physical/chemical/biological): Stability depends on membrane or capsid composition. VLP protein shells are often thermally stable under appropriate ionic/pH conditions and can be stabilized via crosslinking or lyophilization; OMVs retain membrane integrity at physiological temperatures but are subject to lipid oxidation and protein degradation risks [29]. Archaea particles exhibit superior stability due to ether‐linked lipids.

Surface charge and protein corona: Zeta potential affects plasma protein adsorption (protein corona formation), electrostatic interactions, and membrane fusion. Negative surface charge can prolong circulation but may reduce membrane interactions; neutral or slight positive charge enhances cell association but increases nonspecific binding and potential toxicity [161]. Microbial particles present numerous reactive groups (amines, carboxyls, thiols, phosphates) amenable to conjugation.

Modifiability: Native particles offer biological reactive handles (lysine/thiol residues on outer membrane proteins, glycosylation sites) for chemical functionalization via EDC/NHS coupling, maleimide chemistry, or click reactions. Genetic tags like SpyTag/SpyCatcher or aldehyde tags enable controlled covalent coupling for hierarchical functionalization [162].

Design tradeoffs between stability and degradability must be carefully managed: particles should remain stable during circulation yet be stimuli‐responsive at the target to release payloads. Therefore, many engineering approaches adopt “stable exterior with degradable interior” architectures.

### 3.4 Engineering Potential: Genetic Engineering, Chemical Modification, and Surface Antigen or Ligand Display

Engineering transforms native microbial particles into clinically relevant platforms. Engineering strategies fall into genetic, chemical, and modular/combination approaches.

**FIGURE 5:**
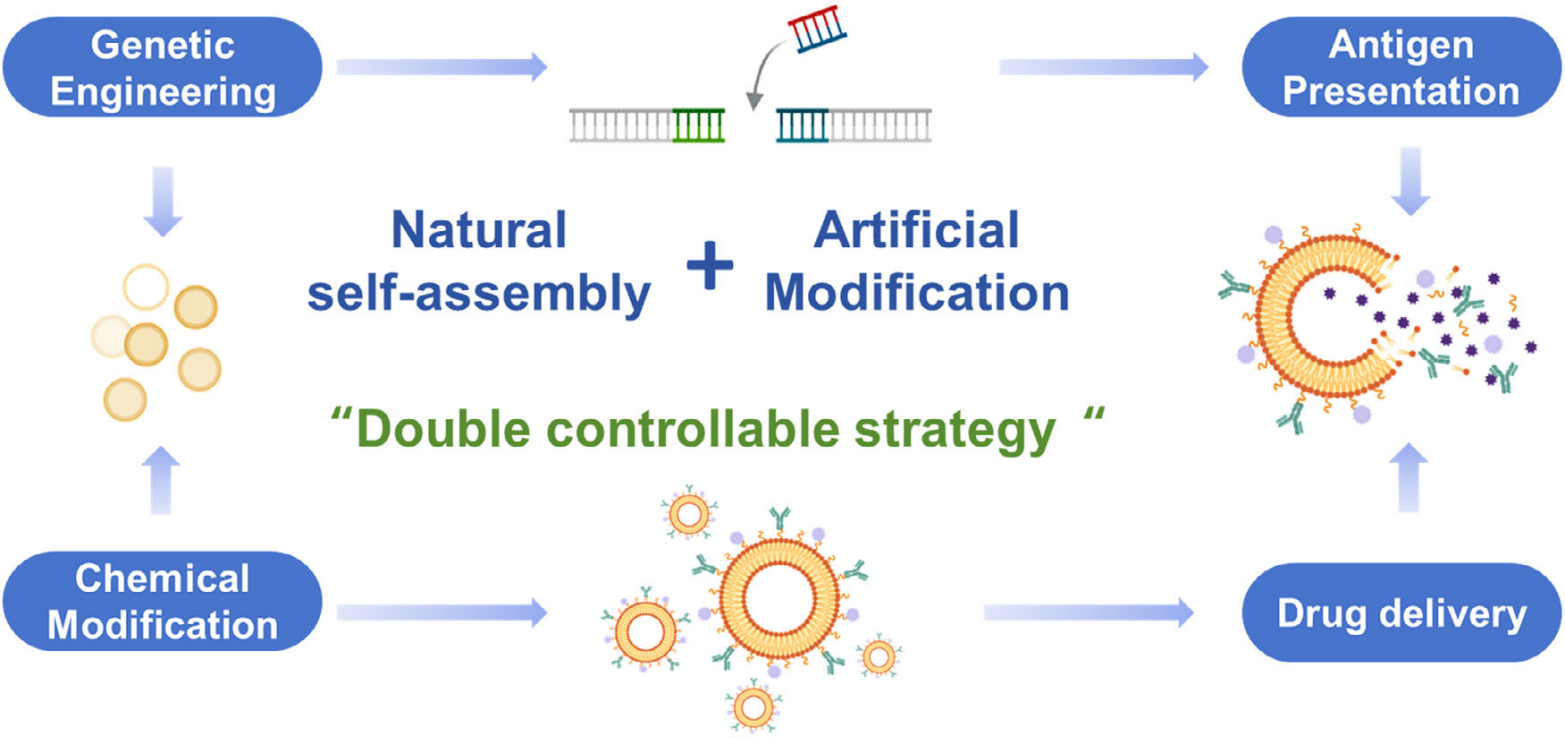
Dual‐controllable engineering strategy for microbial‐derived particles. This figure illustrates a “Natural self‐assembly + Artificial modification” dual‐controllable strategy. Through genetic engineering, outer membrane anchor proteins (such as ClyA, OmpA, and Lpp) or functional fusion genes are introduced to enable natural self‐assembly of particles, facilitating antigen presentation or drug delivery on their surfaces. Subsequently, chemical modification is employed—such as surface conjugation of fluorescent probes, targeting ligands, or therapeutic molecules—to endow the particles with programmable functionalities. This double controllable strategy enables multidimensional regulation from the structural level (assembly morphology) to the functional level (target recognition and responsive release). It enhances the flexibility and controllability of microbial‐derived particles (e.g., OMVs, VLPs, and IBs) in applications such as antigen presentation, immune activation, and precise delivery, offering a rational framework for next‐generation vaccine and biotherapeutic platform design.

Genetic engineering: Molecular cloning and genome editing enable expression of fusion proteins that anchor functional moieties to the particle surface or lumen (Figure 5). Common tactics include fusing antigens or targeting peptides to outer membrane anchors (ClyA, OmpA, Lpp) or inserting epitopes into exposed sites of capsid proteins [163]. CRISPR/Cas and metabolic engineering tools can attenuate endotoxin biosynthesis (e.g., msbB, lpxM knockouts) or upregulate beneficial membrane proteins, optimizing safety and function [164, 165, 166, 167].

Chemical modification: Conjugation chemistries allow grafting of small‐molecule drugs, fluorophores, PEG, or antibody fragments to particle surfaces for targeting, stealth, or tracking. EDC/NHS amide coupling, maleimide–thiol chemistry, and copper‐catalyzed or copper‐free click reactions are widely used [168]. Orthogonal bio‐orthogonal handles (azide/alkyne) enable multi‐component, site‐specific assembly without compromising structural integrity.

Antigen/ligand display: Display may be genetic (encoded fusion) or chemical (site‐specific conjugation). Display density, valency, and spatial arrangement critically influence immunogenicity and receptor binding avidity. High‐density, repetitive antigen arrays on VLPs or OMVs profoundly enhance B‐cell activation and antibody titers; tumor targeting employs small peptide ligands (RGD, NGR) or antibody fragments to drive selective uptake.

Programmability and responsiveness: Synthetic biology elements (inducible promoters, sensor–actuator circuits) permit conditional particle functions. For example, circuits responsive to tumor acidic pH or oxidative stress can trigger local release of immunomodulators, minimizing systemic toxicity.

Manufacturing and quality control are essential for clinical translation. This requires scalable production through fermentation, reproducible purification using techniques like tangential flow filtration, density gradients, and chromatography, along with stringent quality control measures—including assessment of endotoxin levels, size distribution, and proteomic profiles. Robust process parameters and batch‐to‐batch consistency are prerequisites for regulatory approval.

### 3.5 Advanced Imaging Techniques for MDP Manufacturing and Characterization

Advanced imaging technologies play a pivotal role in elucidating the biogenesis, structural heterogeneity, and functional integrity of microbe‐derived particles, thereby bridging fundamental biological understanding with scalable manufacturing and translational quality control [19]. While conventional bulk characterization methods (e.g., DLS, NTA, proteomics) provide averaged information, high‐resolution imaging techniques uniquely enable direct visualization of particle morphology, assembly pathways, and cargo organization at the single‐particle level—features that are essential for both mechanistic insight and regulatory‐grade evaluation [21].

Electron microscopy (EM) remains the cornerstone for structural characterization of MDPs. Transmission electron microscopy (TEM) and scanning electron microscopy (SEM) have been extensively employed to visualize the size, shape, and surface topology of outer membrane vesicles (OMVs), inclusion bodies (IBs), virus‐like particles (VLPs), and polysaccharide‐based particles [169]. TEM readily reveals vesicle integrity, membrane thickness, and multilamellarity, while SEM provides complementary information on surface roughness and aggregation states, particularly for IBs and extracellular polysaccharide particles. However, conventional EM sample preparation may introduce dehydration‐ or staining‐induced artifacts, limiting accurate structural interpretation.

To overcome these limitations, cryo‐electron microscopy (cryo‐EM) and cryo‐electron tomography (cryo‐ET) have emerged as transformative tools for native‐state visualization. Cryo‐EM preserves hydrated MDPs in vitreous ice, enabling near‐physiological observation of membrane curvature, bilayer organization, and capsid symmetry without chemical fixation [170]. Cryo‐ET further enables 3D reconstruction of MDPs in situ, providing unprecedented insight into OMV budding processes, multilayered vesicle formation, and cargo distribution across membrane compartments [171]. For VLPs, single‐particle cryo‐EM has achieved near‐atomic resolution, allowing precise assessment of capsid assembly fidelity, symmetry defects, and epitope display density—parameters that directly correlate with immunogenic performance.

Beyond electron‐based techniques, atomic force microscopy (AFM) offers unique advantages in probing the nanomechanical and surface properties of MDPs under near‐physiological conditions [172]. AFM enables measurement of particle stiffness, elasticity, and rupture forces, which are particularly relevant for IBs, β‐glucan particles, and archaeal vesicles with exceptional mechanical stability. Such mechanical fingerprints provide complementary quality attributes that are not accessible through optical or electron microscopy and are increasingly recognized as indicators of batch consistency and functional robustness.

Advanced fluorescence and super‐resolution microscopy further expand the imaging toolbox for MDP analysis. Confocal and total internal reflection fluorescence (TIRF) microscopy enable spatial tracking of fluorescently labeled cargos, surface ligands, or targeting moieties on engineered MDPs [173]. More recently, super‐resolution techniques such as STED, PALM, and STORM have broken the diffraction limit, allowing nanoscale mapping of protein or nucleic acid distribution on vesicle membranes. These approaches are particularly powerful for validating genetic or chemical engineering strategies, confirming surface display efficiency, and resolving heterogeneity within engineered particle populations.

Importantly, imaging techniques are increasingly integrated into manufacturing workflows and quality control (QC) frameworks. High‐throughput cryo‐EM screening, automated image analysis, and AI‐assisted particle classification enable statistical assessment of size distribution, structural integrity, and assembly defects across production batches [174]. When combined with process analytical technologies (PAT), imaging‐guided feedback can inform optimization of culture conditions, induction parameters, and purification strategies, thereby improving yield reproducibility and reducing batch‐to‐batch variability. Such capabilities are critical for meeting regulatory expectations in clinical translation, where structural consistency and product identity are paramount.

In summary, advanced imaging technologies are not merely descriptive tools but constitute an essential pillar for the rational manufacturing, precise characterization, and translational validation of MDP‐based platforms. The continued convergence of high‐resolution imaging, automated analysis, and process engineering is expected to accelerate the maturation of MDPs from biologically inspired constructs into standardized, regulatory‐compliant nanomedicine products.

## 4 Applications of Microbe‐Derived Particles

### 4.1 Therapeutic Applications

#### 4.1.1 Cancer Therapy

**FIGURE 6:**
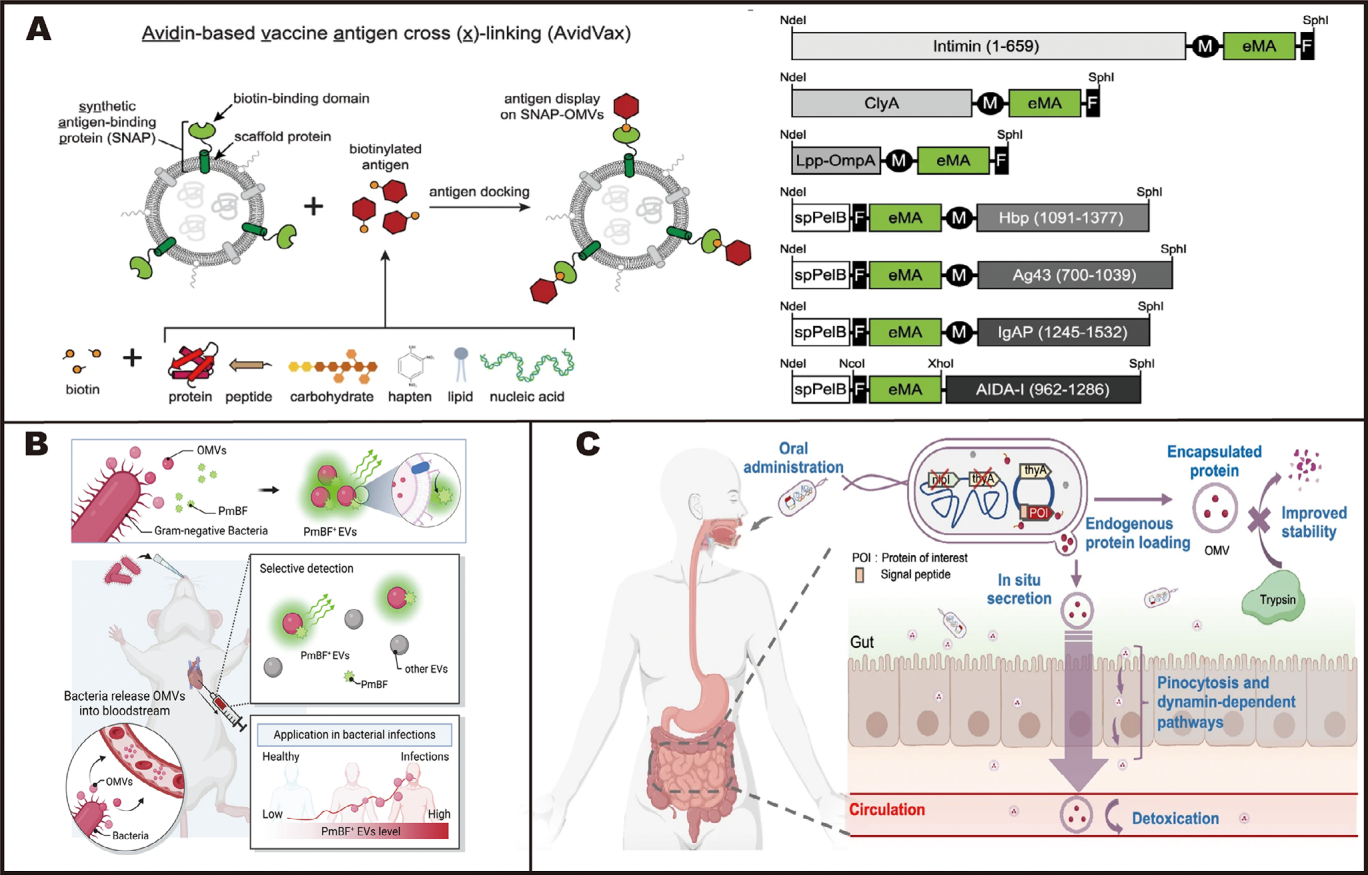
(A) A modular platform for rapid self‐assembly of OMV‐based vaccine candidates. Reproduced with permission. [179] Copyright 2023, Springer Nature. (B) Specific Labeling of Outer Membrane Vesicles with Antibiotic‐conjugated Probe Reveals Early Bacterial Infections in Blood.Reproduced with permission. [176] Copyright 2025, Springer Nature. (C) Schematic of the oral delivery of therapeutic protein by synthetic commensal bacteria outfitted with modified T0SS to detoxify circulating metabolites. Reproduced with permission. [178] Copyright 2025, Springer Nature.

Among all therapeutic applications, the use of MDPs in tumor immunotherapy has been most extensively studied. OMVs and VLPs, owing to their abundant PAMPs and multivalent antigen presentation capability, can efficiently elicit both innate and adaptive immune responses. Natural OMVs can activate dendritic cells (DCs) and macrophages through Toll‐like receptor (TLR) signaling pathways, inducing the secretion of inflammatory cytokines such as IL‐12 and TNF‐α, thereby promoting CD8^+^ T cell‐mediated tumor clearance [23].To reduce toxicity and enhance specificity, engineered OMVs have been developed to display tumor‐associated antigens (TAAs) such as MUC1, HER2, and OVA through genetic fusion or chemical conjugation, enabling precise immune activation. For example, Nie et al. demonstrated that engineered OMVs, displaying tumor‐associated antigens (proteins or peptides) on their surface, effectively elicit antigen‐specific T cell responses and suppress tumor growth and metastasis [175]. In the realm of diagnostics, the PmBF probe technology developed by Li et al. enables the highly specific detection of bacterial OMVs in blood, providing a detectable signal at the very early stages of infection (e.g., within 6 h in a murine pneumonia model) and effectively distinguishing bacterial from viral or mycoplasmal infections, thereby highlighting its promise as an early and precise diagnostic biomarker [176, 177]. For therapeutic delivery, the work by Gong et al. demonstrates the feasibility of using engineered probiotic bacteria as in vivo “cell factories” to encapsulate therapeutic enzymes, such as urate oxidase, within OMVs, achieving successful oral delivery and translocation across the intestinal barrier, which showed significant efficacy in disease models like hyperuricemia and validates OMVs as a viable platform for oral biologics delivery [178]. In vaccine development, the AvidVax platform engineered by Weyant et al., which utilizes a biotin‐avidin system for the rapid and modular display of antigens on the OMV surface, elicits robust antigen‐specific immune responses and offers a versatile strategy for creating vaccines against a broad spectrum of pathogens [179]. Collectively, these applications—spanning diagnosis, therapy, and prevention—underscore the remarkable versatility and significant clinical translation potential of OMVs in biomedical engineering (Figure 6).

Meanwhile, VLPs, with their highly controllable protein self‐assembly capacity, have been widely used in cancer vaccine and immunoadjuvant development. Researchers have exploited VLP scaffolds such as hepatitis B core (HBc), Qβ, and MS2 to display tumor antigens or immunomodulatory molecules, which significantly enhance both antibody titers and T cell responses [180]. For example, HBc VLPs have been used to present tumor neoantigens via a pluganddisplay system, resulting in enhanced dendritic cell maturation, activation of CD4^+^ and CD8^+^ T cells, increased tumor infiltration, and suppression of tumor growth in mouse xenograft models [181]. Some VLPs can simultaneously encapsulate CpG oligonucleotides or siRNAs, forming multifunctional systems for the co‐delivery of “antigen + adjuvant” [182]. In addition, IBs—solid protein nanoparticles formed by recombinant expression in bacteria—show unique advantages in tumor therapy. Their aggregated protein form enables a sustained‐release effect, prolonging antigen half‐life while maintaining localized immune activation and minimizing systemic toxicity [183, 184, 185].

Collectively, these findings demonstrate that MDPs have evolved from simple immune adjuvants into a new generation of “immune nanofactories,” offering versatile and programmable platforms for cancer immunotherapy.

#### 4.1.2 Antibacterial Therapy: Phage‐ and Vesicle‐Based Antimicrobial Strategies

The growing threat of antibiotic resistance has driven renewed interest in alternative antibacterial strategies based on bacteriophages and bacterial membrane vesicles. Bacteriophage particles specifically recognize bacterial surface receptors and induce targeted lysis without disrupting the host microbiota [186, 187, 188, 189]. In contrast, OMVs act as natural *“antimicrobial nanomissiles,”* carrying active biomolecules such as lysozymes, antimicrobial peptides (AMPs), and DNases that mediate inter‐microbial competition and pathogen inhibition. These nanosized vesicles can fuse with bacterial membranes or deliver their enzymatic cargo directly into recipient cells, thereby exerting potent bactericidal effects [190]. Recent advances in engineered OMVs have enabled the directed loading of specific antibacterial agents—including endolysins, AMPs, and even CRISPR‐Cas systems—to achieve gene‐level bacterial eradication [21, 191]. Compared with conventional antibiotics, such OMV‐ or phage‐based platforms exhibit several advantages, including biofilm penetration, sustained release, and low cytotoxicity, offering promising avenues to combat multidrug‐resistant “superbugs.”

#### 4.1.3 Immunomodulation: Engineered Particles for Cytokine, Nucleic Acid, or Therapeutic Protein Delivery

**FIGURE 7:**
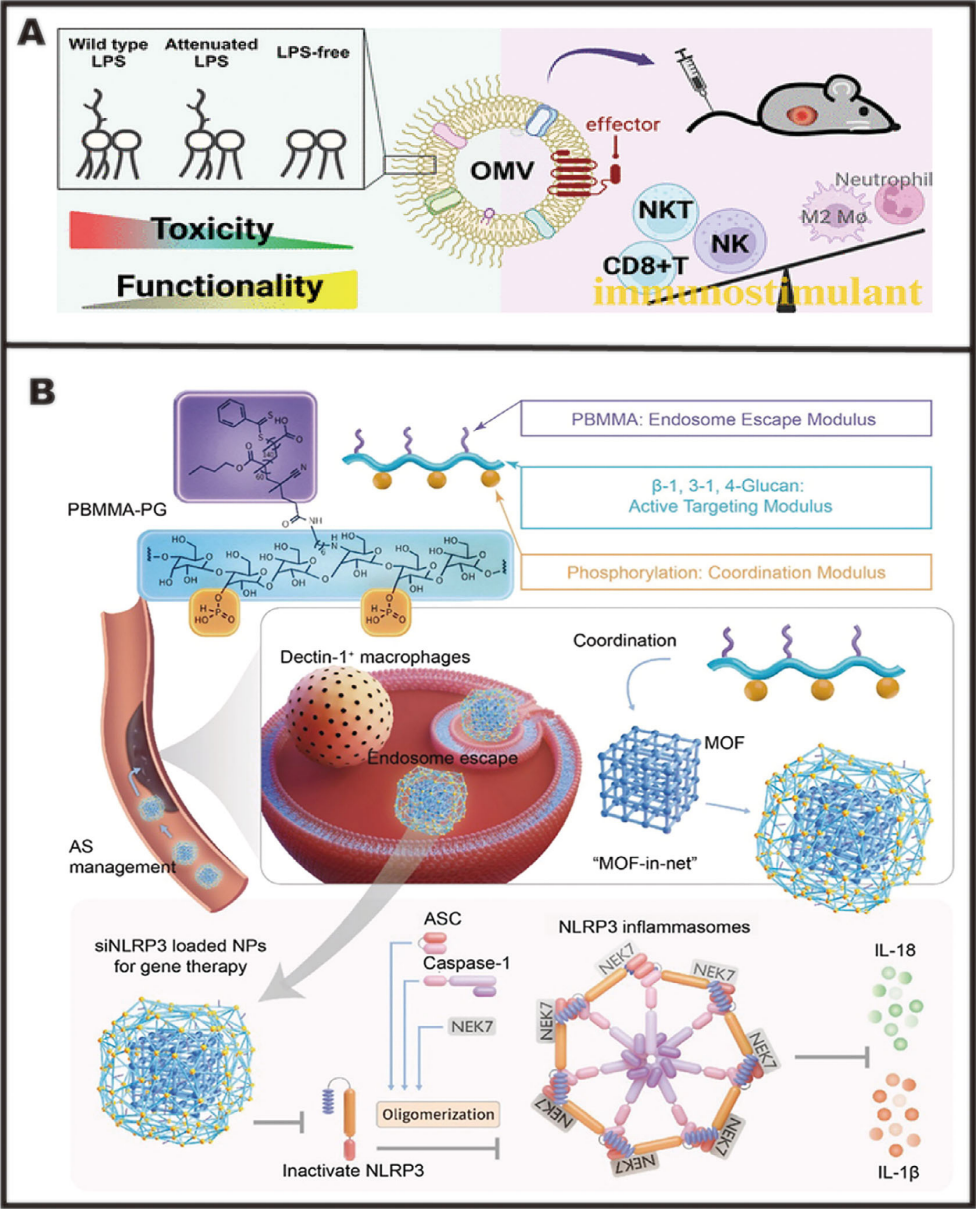
(A) Engineering OMVs as an engineered platform for anti‐cancer immunotherapy. Reproduced with permission. [195] Copyright 2025, American Chemical Society. (B) β‐glucan particles can serve as a ‘Trojan horse’ to specifically deliver immunomodulatory payloads. Reproduced with permission. [194] Copyright 2025, Elsevier.

Beyond direct therapeutic use, MDPs also exhibit remarkable potential in immune modulation. OMVs, BGPs, and IBs can serve as versatile delivery platforms for the targeted delivery of cytokines (e.g., IL‐15 and IL‐27), immunomodulatory proteins, or nucleic acid therapeutics to tumor or inflammatory sites. For example (Figure 7), a recent study by Chen et al. functionalized the IL‑2 variant Neo‑2/15 onto detoxified engineered OMVs, achieving targeted delivery in a mouse tumor model, which enhanced lymphocyte tumor infiltration and strengthened the antitumor immune response, demonstrating the feasibility and potential of this nanodelivery strategy [192].

Among them, β‐glucan particles are efficiently internalized by macrophages via the Dectin‐1 receptor, leading to NF‐κB pathway activation and the subsequent secretion of IL‐12, thereby enhancing both antiviral and antitumor immune responses [193]. These immunostimulatory effects have made β‐glucan particles promising adjuvants and delivery carriers in immune‐oncology. For instance, a recent advanced application demonstrated that β‐glucan particles can serve as a ‘Trojan horse’ to specifically deliver immunomodulatory payloads (e.g., TLR agonists or mRNA encoding IL‐12) to tumor‐associated macrophages via Dectin‐1 targeting. This strategy effectively reprogrammed immunosuppressive M2 macrophages toward an antitumor M1 phenotype, potentiated IL‐12 secretion, and elicited robust systemic antitumor immunity in murine models, highlighting their great potential as sophisticated delivery platforms in cancer immunotherapy [194].

Moreover, fungal extracellular vesicles (EVs), which are rich in signaling RNAs, lipids, and immunomodulatory molecules, can modulate host immune responses by inducing immune remodeling or tolerance. Such vesicles have demonstrated potential in regulating immune homeostasis during infectious diseases and autoimmune disorders [195, 196, 197, 198].

### 4.2 Diagnostics and Imaging: Biomarker and Imaging Potential of Microbe‐Derived Particles

#### 4.2.1 Microbial Vesicles as Biomarkers for Liquid Biopsy

Microbial vesicles are selectively released during infection and within tumor microenvironments, and their molecular cargo—including proteins, RNAs, and lipids—can reflect the physiological or pathological state of the host and pathogen. Owing to their stability and detectability, bacterial OMVs and fungal extracellular vesicles (EVs) have emerged as promising biomarkers for liquid biopsy–based diagnostics. For instance, Helicobacter pylori–derived OMVs contain virulence proteins such as VacA [199] and CagA [200], which can be internalized by gastric epithelial cells to deliver these proteins, thereby promoting inflammation, genomic instability, and carcinogenic processes. Although not yet clinically validated, the presence of CagA and VacA within H. pylori OMVs suggests their potential as biomarkers for the early detection of gastric cancer or H. pylori–associated precancerous lesions [201, 202].

Similarly, Candida‐derived EVs exhibit distinct proteomic profiles that can be used to differentiate invasive fungal infections from colonization or non‐pathogenic states [203]. These findings suggest that analysis of microbe‐derived vesicles in body fluids could enable noninvasive diagnostics and real‐time disease monitoring through molecular profiling.

#### 4.2.2 Engineered VLPs as Imaging Probes and Signal Amplification Platforms

Through fluorescent labeling, magnetic nanoparticle conjugation, or metal chelation modification, VLPs and OMVs can be engineered as molecular imaging probes for MRI, PET, or photoacoustic imaging [180, 204]. Their intrinsic nanoscale dimensions, monodispersity, and highly modifiable surface chemistry confer superior circulatory stability and signal amplification compared with many synthetic nanoparticles. For example, plantvirus‐derived VLPs (e.g., PhMV VLPs) have been chemically functionalized with fluorescent dyes for robust cellular uptake and fluorescence imaging of cancer cells; moreover, VLPs carrying MRI contrast agents have exhibited significantly enhanced relaxivity in vitro and in vivo, highlighting VLPs as a versatile platform for molecular imaging and theranostics [205, 206]. Collectively, these strategies highlight the potential of MDPs as biocompatible and multifunctional imaging carriers for disease detection and monitoring.

### 4.3 Drug Delivery and Biotechnological Applications

#### 4.3.1 Nucleic Acid and Protein Delivery Platforms

Due to their membrane‐enclosed structure and self‐assembly capabilities, OMVs and VLPs have emerged as ideal carriers for the delivery of RNA, proteins, and small‐molecule therapeutics. Engineered OMVs can be functionalized through fusion of outer membrane proteins to encapsulate mRNA, siRNA, or CRISPR‐Cas components, which are subsequently released into target cells, enabling intracellular genetic modulation. Similarly, VLPs, owing to their modular architecture and programmable assembly, allow for precise control over cargo loading and release kinetics. Recent studies have demonstrated that VLPs can successfully deliver CRISPR–Cas9 nucleic acid systems, achieving efficient in vivo gene editing [207]. More recently, programmable VLPs were used to deliver Cas9 RNPs for ocular neovascularization and Huntington's disease models, achieving targeted in vivo gene editing and therapeutic effects [208]

#### 4.3.2 Protein Sustained Release and Oral Delivery Systems

IBs are naturally occurring protein aggregates with remarkable stability and slow‐release characteristics. Their solid‐state structure permits gradual protein release while preventing proteolytic degradation, making them valuable for long‐term protein drug delivery applications [209]. In contrast, β‐glucan particles (BGPs) are resistant to gastric acid and can protect encapsulated therapeutics from enzymatic digestion during oral administration [81, 210, 211]. By facilitating intestinal targeting and mucosal absorption, β‐glucan particles are considered a representative class of “edible delivery vehicles” with potential applications in oral immunotherapy and nutraceutical formulations.

In addition to their performance in conventional biomedical and biotechnological applications, certain microbe‐derived particles (MDPs) exhibit enhanced structural robustness under harsh physicochemical conditions, including elevated temperature, high salinity, extreme pH, and oxidative stress. This intrinsic stability, often arising from their microbial origin and unique assembly architectures, has prompted exploratory investigations into niche applications where conventional synthetic nanomaterials or biologics rapidly lose functionality.

For example, proteinaceous inclusion bodies and archaeal‐derived vesicles have demonstrated remarkable resistance to thermal and chemical denaturation, enabling their use as immobilized biocatalysts or enzyme carriers in non‐physiological reaction environments. Similarly, polysaccharide‐based MDPs, such as β‐glucan particles, maintain structural integrity under acidic or enzymatically aggressive conditions, supporting their application in environmental sensing and delivery contexts. Notably, in these scenarios, the advantage of MDPs lies less in novel functionality than in their ability to preserve bioactivity and structural coherence under conditions that challenge conventional delivery platforms.

Nevertheless, it should be emphasized that such applications remain limited in scope and are largely at a proof‐of‐concept stage. Current studies predominantly highlight stability‐related advantages rather than well‐established, large‐scale deployment. As such, MDP utilization under extreme conditions is best viewed as an emerging extension of their broader application landscape, rather than a standalone application domain.

MDPs have shown broad potential in vaccines, drug delivery, immunomodulation, and environmental sensing. Their intrinsic bioactivity and engineering flexibility offer clear advantages, yet challenges remain. Consistent dosing, tunable immunogenicity, and scalable production continue to limit translational feasibility. Careful choice of particle type, engineering strategy, and delivery modality is therefore critical to balance efficacy, safety, and manufacturability in clinical and industrial contexts.

## 5 Advantages and Challenges

MDPs, including bacterial outer membrane vesicles and other nanoscale microbial assemblies, have attracted increasing attention in biomedical research due to their unique structural features and multifunctional bioactivity. Notably, certain subclasses of MDPs demonstrate favorable scalability, as they can be produced through established microbial fermentation platforms combined with genetic modulation and controlled induction strategies, which facilitate improved batch consistency and translational feasibility [29]. In addition, the immunogenicity of MDPs is highly tunable, allowing precise modulation of host immune responses. Genetic engineering can be employed to modify or remove specific immunostimulatory components, such as lipopolysaccharides (LPS) in Gram‐negative bacterial OMVs, or to introduce genes encoding desired antigens or immune‐regulatory proteins. Complementary chemical strategies—including LPS detoxification, selective proteolysis, or surface conjugation with polymers or targeting ligands—further minimize undesired systemic inflammation while preserving or enhancing antigen‐specific immune activation. Together, these approaches allow MDPs to achieve a controlled balance between safety and efficacy, tailoring their immunogenic profiles for applications such as vaccination, cancer immunotherapy, or immune modulation [212]. Moreover, MDPs demonstrate cross‐species applicability and excellent biocompatibility, capable of interacting with diverse host systems, traversing biological barriers, and serving as delivery vehicles for antigens, RNAs, or metabolites, thereby enabling multiple functional applications. These characteristics collectively render MDPs highly versatile platforms for vaccine development, drug delivery, and immunomodulation.

Nevertheless, the very properties that confer versatility also introduce significant translational complexity. Despite their promise, the clinical development of MDPs remains constrained not only by classical concerns of biosafety and purification, but also by deeper conceptual and mechanistic challenges. Unlike fully synthetic nanoparticles, MDPs retain intrinsic biological cues—including PAMPs, membrane proteins, and endogenous bioactive cargo—that may interact with host immunity in nonlinear and context‐dependent manners [213, 214, 215]. In addition, despite improvements in manufacturing control, standardized criteria for defining functional potency and biological batch equivalence are still lacking. Without validated structure–function correlations and robust quality control metrics, it is difficult to guarantee that batches with similar physicochemical properties will consistently produce comparable biological outcomes [216]. Looking forward, advancing MDP‐based therapeutics may require a conceptual shift—from viewing these particles as naturally derived carriers to developing programmable, modular bio‐nanoplatforms with defined composition and controllable bioactivity. Integration of synthetic biology, quantitative omics, and predictive modeling will be critical to reducing functional uncertainty and establishing reproducible translational pipelines. Addressing these mechanistic and regulatory dimensions in parallel will ultimately determine whether MDPs transition from promising experimental systems into clinically viable platforms.

## 6 Perspectives

MDPs, as an emerging bio‐nanoplatform, hold substantial potential for future development, particularly driven by the integration of synthetic biology, materials science, and immunology [19]. Through this interdisciplinary approach, researchers can precisely engineer particle composition and bioactivity within microbial hosts, while leveraging materials science to impart multifunctional payloads, environmental responsiveness, and intelligent release capabilities, and applying immunological strategies to optimize immunogenicity and safety. This enables MDPs to achieve efficient targeting of specific cells or tissues and spatiotemporal delivery of drugs, immunomodulatory molecules, or signaling factors, while multifunctional engineering supports combined therapies, immune regulation, and diagnostic monitoring [217, 218].

Despite these promising prospects, clinical translation of MDPs faces challenges such as scalable production, compositional complexity, tunable immunogenicity, potential toxicity, and cross‐species applicability [219]. Future strategies may address these hurdles through standardized manufacturing and purification processes, precise component design, high‐throughput screening, and nanoparticle engineering, while developing products that are mass‐producible, batch‐consistent, and easily tunable, thereby laying the foundation for clinical and industrial applications [220]. Based on these technological advances, MDPs are expected to play important roles in cancer immunotherapy, microbiome modulation, and smart biomaterials development, as well as serving as efficient carriers for vaccines or diagnostic tools, providing innovative and scalable strategies for future precision biomedicine [221].

## 7 Conclusion

Overall, MDPs combine natural origin, compositional diversity, engineerability, and cross‐species functionality, highlighting their unique value in diagnostics, therapeutics, and biotechnology. Their highly tunable targeting, immune modulation, and controllable release capabilities provide novel platforms for personalized therapies and precision diagnostics. With ongoing advances in production techniques and interdisciplinary research, MDPs are poised to become emerging tools in future biomedicine and biotechnology, offering scalable, tunable, and safe strategies. This underscores the rapidly growing potential of microbial particle technology in life sciences and precision medicine.

## Conflicts of Interest

The authors declare no conflicts of interest.
